# Sigmoid colon schwannoma difficult to distinguish from peritoneal dissemination 13 years after pancreatic neuroendocrine tumor surgery

**DOI:** 10.1186/s40792-023-01658-z

**Published:** 2023-05-11

**Authors:** Kazunobu Suzuki, Takuya Shiraishi, Ikuma Shioi, Naoya Ozawa, Takuhisa Okada, Katsuya Osone, Takaaki Sano, Kenichiro Araki, Hiroomi Ogawa, Akihiko Sano, Makoto Sakai, Makoto Sohda, Ken Shirabe, Hiroshi Saeki

**Affiliations:** 1 Department of General Surgical Science, Gunma University Graduate School of Medicine, Maebashi, Japan; 2 Department of Diagnostic Pathology, Gunma University Graduate School of Medicine, Maebashi, Japan

**Keywords:** Laparoscopy, Long-term metastasis, Pancreatic neuroendocrine tumor, Schwannoma

## Abstract

**Background:**

Schwannoma, which clinicians sometimes struggle to diagnose, is a tumor arising from Schwann cells of peripheral nerves, often in the soft tissues and rarely in the gastrointestinal tract. Pancreatic neuroendocrine tumor (PNET) is rare among pancreatic tumors, and recurrence can occur long after resection. Here, we were presented with a case where a sigmoid colon schwannoma was difficult to distinguish from a postoperative recurrence of PNET and was diagnosed after laparoscopic resection.

**Case presentation:**

A 51-year-old man was diagnosed with PNET (NET G2) after a distal pancreatectomy (DP) 13 years ago. The patient underwent hepatectomy due to liver metastasis 12 years after initial radical surgery. The follow-up magnetic resonance imaging (MRI) after hepatectomy showed pelvic nodules, and laparoscopic surgery was performed for both diagnosis and treatment because peritoneal dissemination of PNET could not be ruled out. Since the tumor was in the sigmoid colon, a partial colon resection was performed. The histopathological diagnosis was a schwannoma, and the patient was discharged on the seventh postoperative day.

**Conclusions:**

We experienced a case of sigmoid colon schwannoma that was difficult to differentiate from peritoneal dissemination of PNET and was later diagnosed after laparoscopic resection. In addition, this case involved a long-term postoperative recurrence of PNET that was amenable to radical resection, further establishing the importance of long-term imaging follow-up.

## Background

Schwannoma is a tumor arising from Schwann cells of peripheral nerves, mostly occurring in soft tissues and less commonly in the gastrointestinal tract. Gastrointestinal schwannomas (GIS) arise from the Meissner or Auerbach plexus. GIS accounts for 2–6% of all stromal tumors in the gastrointestinal tract [1] and is most common in the stomach and relatively rare in the colon. Although differential diagnoses for GIS include gastrointestinal stromal tumors (GIST), neuroendocrine tumors (NET), leiomyoma, leiomyosarcoma, and adenocarcinoma, it may be difficult to diagnose them preoperatively, even with the use of multiple diagnostic modalities.

Pancreatic neuroendocrine tumors (PNETs), also known as islet cell tumors, are relatively rare among pancreatic tumors, with a 5-year overall survival rate of 53% [2]. The basic treatment for PNET is radical surgery. Unfortunately, PNETs have a recurrence rate of 15% in various organs after surgery, including the peritoneum, and should be followed up diligently as recurrences could occur even 10 years after the primary malignancy [3]. Here, we report a case of sigmoid colon schwannoma that was difficult to distinguish from peritoneal dissemination after radical resection for multiple liver metastases 12 years after the diagnosis of PNET. The case was diagnosed after laparoscopic resection.

## Case presentation

**Fig. 1:**
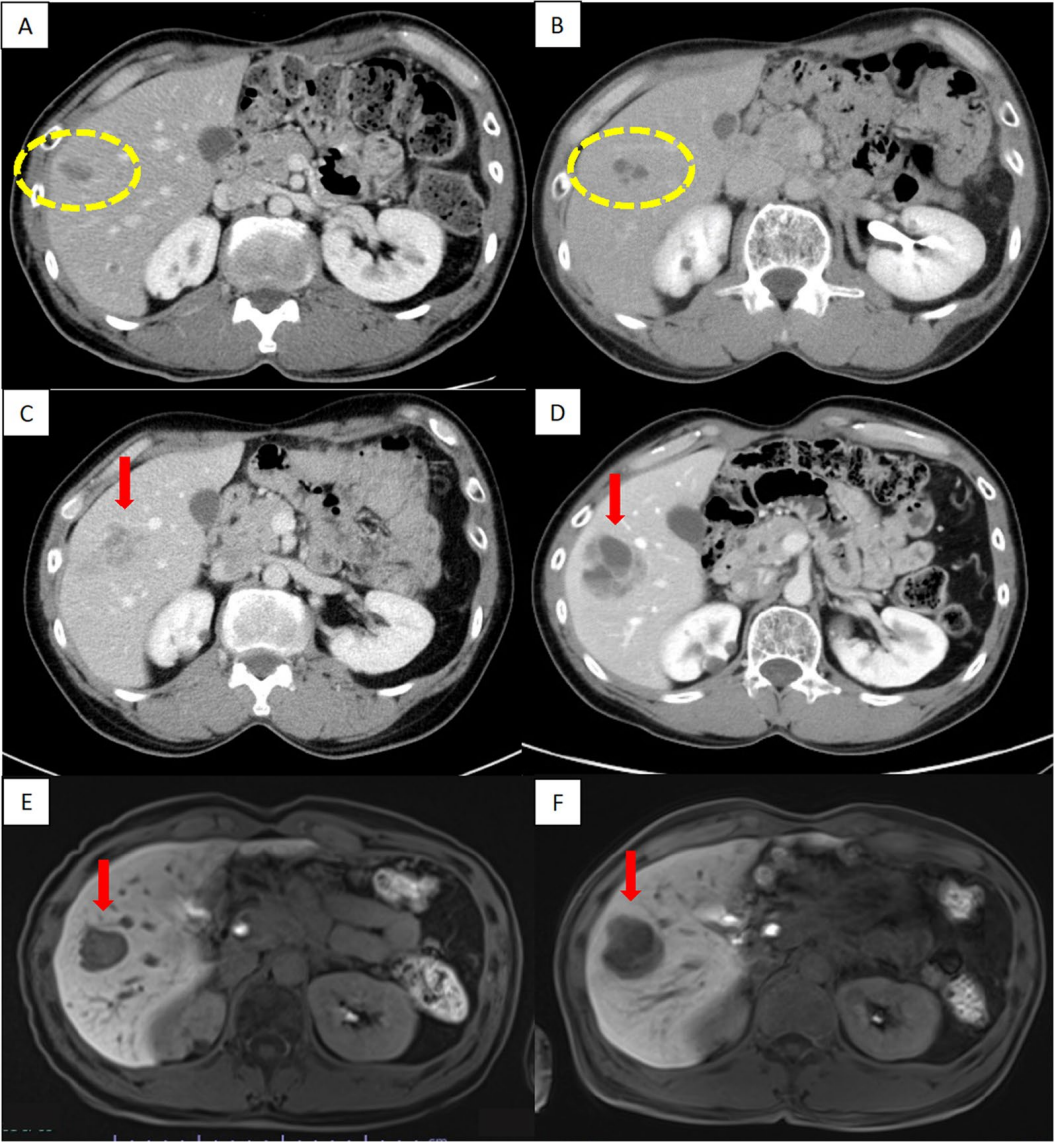
Enlargement of PNET liver metastasis during follow-up. **A**, **B** There was no obvious tendency toward enlargement at 6 months (**A**) and 6 years (**B**) after the initial surgery (circle). The greatest diameter increased from 23 to 36 mm after one year (arrow). **C**, **D** Contrast-enhanced CT scan. **E, F** Contrast-enhanced MRI (T1-weighted with fat suppression). *CT* computed tomography, *MRI* magnetic resonance imaging

**Fig. 2:**
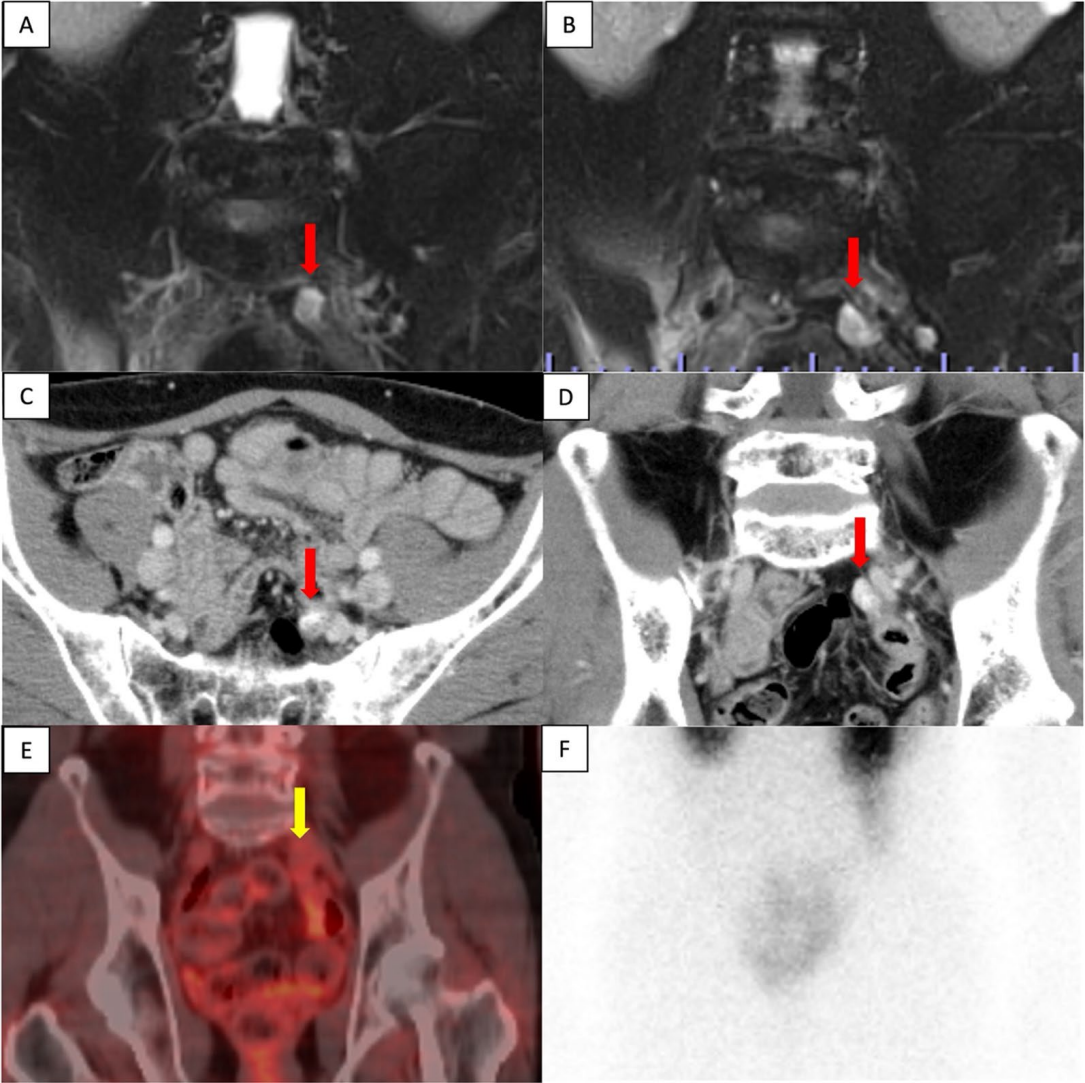
A nodular lesion in the pelvis (arrow). **A**, **B** A contrast-enhanced MRI (T1-weighted with fat suppression) showed an intrapelvic lesion increasing in size from 13 to 19 mm after 4 months. **C**, **D** A contrast-enhanced CT showed a 20-mm nodular lesion with a contrast effect. **E** FDG-PET/CT showed a low accumulation of FDG in the nodular lesion. **F** SRS showed the low expression of somatostatin receptors in the nodular lesion. *CT *computed tomography, *MRI* magnetic resonance imaging, *SRS* somatostatin receptor scintigraphy, *PET* positron-emission tomography, *FDG* fluorodeoxyglucose

**Fig. 3:**
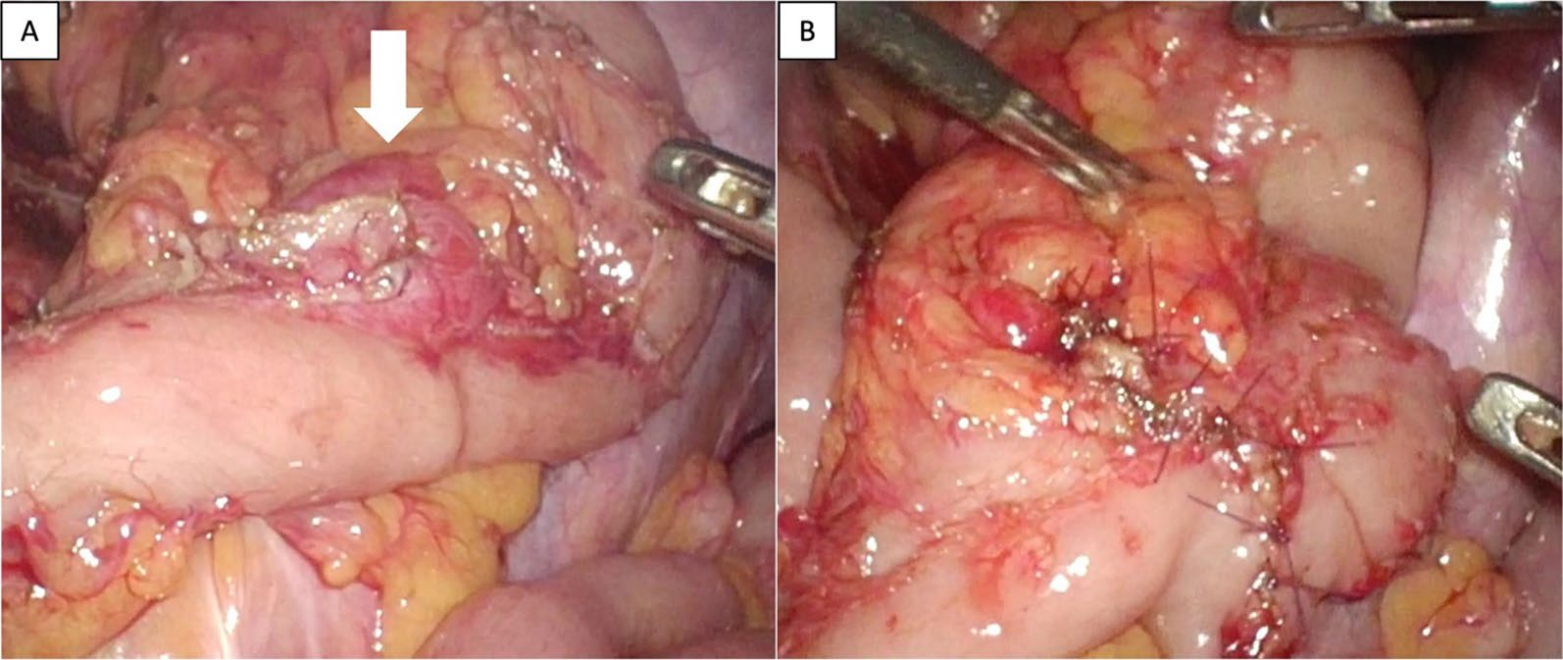
Intraoperative findings. **A** The neoplastic lesion was found in the mesenteric side of the sigmoid colon after the dissection of adhesions around the area (arrow). The lesion involved the mesentery of the sigmoid colon. **B** A partial colectomy was performed, and the defective intestinal wall was sutured

**Fig. 4:**
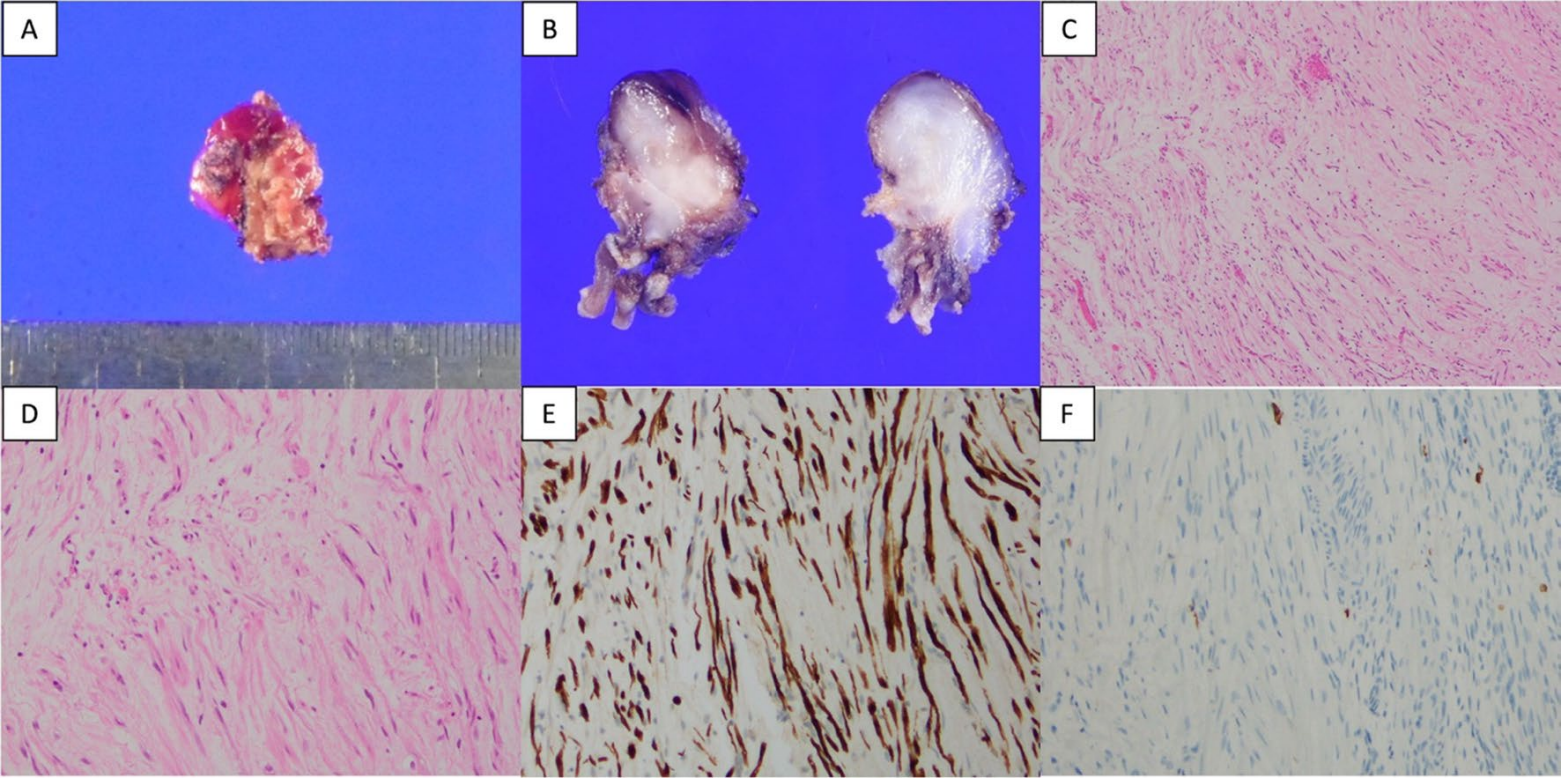
The resected specimen. **A**, **B** The tumor was 30 × 16 mm in size, and the split surface was white. **C**, **D** Microscopic examination showed an arrangement of eosinophilic spindle-shaped cells in the colonic wall (**C**: hematoxylin and eosin × 40, **D**: hematoxylin and eosin × 100). Immunohistochemistry revealed the expression of S100 (**E**) and non-expression of c-kit (**F**)

A 51-year-old man underwent a distal pancreatectomy (DP) for PNET 13 years ago, with a histopathological diagnosis of NET G2. Lymph node metastasis was positive in 1/14 (near the pancreas); endocrine function was insulin negative, glucagon negative, and somatostatin negative; and Ki67 was 4%. A computed tomography (CT) scan showed indications of liver metastasis 6 months after surgery (Fig. 1A); however, as there was no obvious tendency toward enlargement (Fig. 1B) and multiple biopsies showed no evidence of malignancy. Thus the patient was followed up with CT and magnetic resonance imaging (MRI) without additional treatments. Although the patient was followed up for 12 years after DP, CT, and MRI, these studies showed multiple lesions in both liver lobes and a trend toward enlargement with an increase from 23 to 36 mm in greatest diameter after one year (Fig. 1C–F). Left hepatectomy, partial resection, and cholecystectomy were performed for liver metastases. The Ki67 positivity rate of liver metastatic lesions was heterogeneous, ranging from < 3% to 20%. A follow-up MRI taken 1 year after liver surgery showed a nodular lesion in the pelvis with the greatest diameter of 13 mm; a MRI conducted 4 months later showed an enlargement to 19 mm in diameter (Fig. 2). The nodules were not identified during the surgical resection of the liver metastasis. Serum neuron-specific enolase (NSE) was within normal limits, and colonoscopy (CS) findings were unremarkable. An ultrasound endoscopy was not performed because the tumor location could not be identified inside the gastrointestinal tract. A contrast-enhanced CT showed a 20-mm intrapelvic nodule, and ^18^F-fluorodeoxyglucose (FDG)-positron-emission tomography (PET)/CT showed low FDG accumulation in the nodule (Fig. 2). Somatostatin receptor scintigraphy (SRS) showed low expression of somatostatin receptors in the nodule (Fig. 2). The peritoneal dissemination of PNET was also deemed possible. Thus, laparoscopic surgery was performed as a diagnostic and potentially therapeutic procedure. The neoplastic lesion was found in the mesenteric side of the sigmoid colon after the dissection of adhesions surrounding the area. It was difficult to excise the tumor alone because the lesion involved the mesentery of the sigmoid colon. Thus, partial colectomy was performed, and the defective intestinal wall was sutured (Fig. 3). The operative duration was 2 h and 45 min, and there was no blood loss. The tumor was 16 × 13 mm in size, and the split surface was white (Fig. 4). Histopathological examination showed that the tumor originated from sigmoidal muscularis propria on the serosal side and an arrangement of eosinophilic spindle-shaped cells in the colonic wall, and immunohistochemistry revealed expression of S100 but no expression of c-kit (Fig. 4), confirming the diagnosis of schwannoma arising from the Auerbach’s plexus. On the seventh postoperative day, the patient was discharged.

## Discussion

PNETs account for less than 5% of all pancreatic tumors, and although resection is the treatment of choice in principle, PNETs are known to recur frequently after surgery. Dong et al. reported that 154 (15.1%) of 1,020 postoperative patients with PNET experienced recurrence, with the liver (*n* = 76, 49.4%) and pancreas (*n* = 35, 22.7%) being the primary sites of recurrence [3]. In the same paper, it was reported that about half the recurrences occurred within 2 years after surgery but could also occur even after almost 10 years. Similarly, Kim et al. also reported cases of liver metastases recurrence more than 10 years after surgery, suggesting the need for long-term postoperative follow-up [4]. In the present case, liver metastasis was suspected 6 months after surgery, but there were no malignant findings. After continuous follow-up, an increasing trend toward recurrence was observed 12 years later. A lesion that was initially found 6 months post-surgery was later found to be an enlarging neoplasm after 10 years. To date, this has never been reported; this, however, suggests that slow-growing tumors may exist in PNETs even after malignancy has been ruled out.

Careful follow-up is important to avoid missing the optimal timing of resection. Tumor markers should be measured every 3 months, and imaging studies should be performed every 6 months during the first 2 years, and then every year thereafter [5]. It has been reported that follow-up is necessary for at least 10 years because recurrence may occur after a long period [5]. In the present case, recurrence happened more than 10 years after primary resection, and radical resection was still possible with the diagnosis of recurrence. While many cases recur soon after surgery, some recur after a longer timeframe, and careful long-term follow-up should be done to avoid missing the opportunity for resection, especially if recurrence is suspected on the postoperative follow-up examination.

When the development of schwannoma is observed during the postoperative follow-up of a malignant tumor, a definitive diagnosis is difficult. Although differential diagnoses for GIS include GIST, NET, leiomyoma, leiomyosarcoma, and adenocarcinoma, it is difficult to confirm these diagnoses through imaging studies alone. GIS appear as well-defined homogenous tumors with low enhancement on CT compared to the heterogeneous appearance of GIST or the obscure characteristics of adenocarcinoma. However, these features are not highly specific [6]. CT has been reported to have a sensitivity of 61–90%, specificity of 90–92%, and accuracy of 58–90% in the diagnosis of NET metastases [7–10]. It is difficult to distinguish NETs from CTs alone. Unlike highly differentiated NETs, many cases of schwannoma show increased accumulation on FDG-PET/CT. However, it is difficult to distinguish benign from malignant tumors, and it is also not possible to distinguish between GIS and NETs [6]. SRS for NETs is highly specific (93%), but it is neither sensitive (52%), nor accurate (58%) [8]. Additionally, it is not possible to distinguish between GIS and NETs if SRS is negative. Many cases could not be definitively diagnosed even when multiple modalities were used. Therefore, a histopathological examination is required for a definitive diagnosis. In this case, although CT showed findings suspicious for GIS and NET, FDG-PET/CT did not show typical findings of schwannoma, and SRS did not lead to the diagnosis of NET. Imaging alone could not distinguish between peritoneal dissemination of NETs and GIS, and histopathological examination was required.

A histopathological diagnosis of GIS can be made by endoscopy or surgery. Although endoscopic identification is possible in some cases, surgery may be required in cases where endoscopic lesions are not seen or a biopsy is difficult to diagnose [6]. In the present case, no lesion was endoscopically identified, and surgery was necessary for a histopathological diagnosis. Peritoneal dissemination of PNET was suspected because of the gradual increase in tumor size, but no other metastases, invasion into the surrounding tissue, or enlargement of lymph nodes were observed. Thus, the patient underwent laparoscopic surgery for diagnostic and therapeutic purposes. Laparoscopy in bowel surgery has been associated with lower perioperative mortality, lower complication rates, earlier discharge, and lower hospitalization costs compared with open surgery and is therefore considered less invasive [11]. In addition, laparoscopic surgery allows a wider view of the abdominal cavity when adhesions are minimal. CT scans and FDG-PET are useful for detecting peritoneal dissemination, and the combination of both modalities allows for the detection of most lesions; however, their combination may be insufficient for detecting very small lesions. Although adhesions are expected in cases with a history of surgery, it could be possible to search for intra-abdominal lesions by combining preoperative imaging with laparoscopy, which is less invasive and allows for intra-abdominal inspection. Therefore, we selected the laparoscopic approach, ensured radical resection, and achieved a definitive diagnosis with minimal invasiveness.

## Conclusions

We report a case of sigmoid colon schwannoma that was difficult to distinguish from peritoneal dissemination of PNET. Laparoscopic surgery was considered useful as a diagnostic and therapeutic approach as it was minimally invasive. In addition, this case showed that radical surgery was possible for the recurrence of liver metastasis 12 years after the initial surgery for PNET. Long-term follow-up with imaging studies is, therefore, important in such cases.

## Data Availability

All data generated or analyzed during this study are included in this published article.

## Abbreviations

GIS: Gastrointestinal schwannomas
GIST: Gastrointestinal stromal tumors
NET: Neuroendocrine tumor
PNET: Pancreatic neuroendocrine tumor
DP: Distal pancreatomy
CT: Computed tomography
MRI: Magnetic resonance imaging
NSE: Neuron-specific enolase
CS: Colonoscopy
FDG: ^18^F-fluorodeoxyglucose
PET: Positron-emission tomography
SRS: Somatostatin receptor scintigraphy
